# Effects of Health Information Dissemination on User Follows and Likes during COVID-19 Outbreak in China: Data and Content Analysis

**DOI:** 10.3390/ijerph17145081

**Published:** 2020-07-14

**Authors:** Rongyang Ma, Zhaohua Deng, Manli Wu

**Affiliations:** 1 School of Medicine and Health Management, Huazhong University of Science and Technology, Wuhan 430030, China; ryma@hust.edu.cn; 2 School of Journalism and Information Communication, Huazhong University of Science and Technology, Wuhan 430074, China; mlwu@hust.edu.cn

**Keywords:** novel coronavirus, official account, health information dissemination, users’ information behavior, regression model, content analysis, characteristic

## Abstract

*Background*: COVID-19 has greatly attacked China, spreading in the whole world. Articles were posted on many official WeChat accounts to transmit health information about this pandemic. The public also sought related information via social media more frequently. However, little is known about what kinds of information satisfy them better. This study aimed to explore the characteristics of health information dissemination that affected users’ information behavior on WeChat. *Methods*: Two-wave data were collected from the top 200 WeChat official accounts on the Xigua website. The data included the change in the number of followers and the total number of likes on each account in a 7-day period, as well as the number of each type of article and headlines about coronavirus. It was used to developed regression models and conduct content analysis to figure out information characteristics in quantity and content. *Results:* For nonmedical institution accounts in the model, report and story types of articles had positive effects on users’ following behaviors. The number of headlines on coronavirus positively impacts liking behaviors. For medical institution accounts, report and science types had a positive effect, too. In the content analysis, several common characteristics were identified. *Conclusions*: Characteristics in terms of the quantity and content in health information dissemination contribute to users’ information behavior. In terms of the content in the headlines, via coding and word frequency analysis, organizational structure, multimedia applications, and instructions—the common dimension in different articles—composed the common features in information that impacted users’ liking behaviors.

## 1. Introduction

Since the outbreak of the novel coronavirus (COVID-19)-infected pneumonia (NCP) in December 2019, it has quickly spread across the world. The World Health Organization declared the outbreak of COVID-19 as a global public health emergency. More than 7 million cases have been confirmed as of 9 June 2020 [1]. COVID-19 has attracted attention worldwide, and information and discussions about it have been spreading on the Internet, especially on social media. The ubiquity and ease of access make social media a powerful complement to traditional methods in information dissemination [2]. According to a report released by CSM Media Research, 77.3% of Chinese people purposefully seek pandemic information online, and approximately 78.7% use WeChat more frequently during the outbreak than before it occurred [3].

Social media is widely used for disseminating health information [4]. As one of the most popular social platforms in China, WeChat has more than 1.1 billion monthly active users [5] and has become a frequently used information dissemination platform [6]. WeChat contains a specific module called WeChat official account [7], which is a platform operated by institutions, communities, or individuals. WeChat official accounts are widely used to share stories, report news, or disseminate various types of information. Information on these accounts can be posted by anyone, including experts, novices, and even saboteurs [8]. WeChat has changed channels of health information dissemination and manners of obtaining feedback [9]. For instance, evidence-based clinical practice guidelines in medical fields are traditionally spread by publishing in peer-reviewed journals, sending emails or paper notices to physicians, and advertising through news media outlets [10]. However, WeChat official accounts now enable guidelines for COVID-19 to be shared on this platform when they are simultaneously published by health authorities.

In response to the pandemic, people prefer to receive real-time news and instructions on personal protection [3]. As such, many WeChat official accounts have posted articles about NCP. However, different account operators tend to post various types of articles in different numbers. For example, some accounts have reported the number of infected cases every day to keep people informed about the pandemic state. Some accounts have instructed the public to protect themselves. Some accounts have refuted fake news to avoid confusion and inappropriate interventions. Health information posted on these accounts can have a great impact on receivers’ behavior because of its real-time nature and various forms [11]. They can express their appreciation and interest by liking an article or following an account [12]. We found that the number of followers on many official accounts changed dramatically within a week. Meanwhile, the number of likes differs greatly among articles. In this work, we aimed to determine whether and how health information dissemination affected users’ information behavior in terms of following an account and liking a post.

Researchers studied the influence of health information on information behaviors on different social media platforms, such as Facebook and microblogging sites. The findings are shown in Table 1.

However, we found that few researchers concentrated on WeChat in China. The above did not study detailed characteristics in the information. These studies mainly focused on the effect of health information on information behavior. However, during the pandemic, users may concentrate on different types of information, and their reaction to a given information may vary. For example, more people have followed WeChat official accounts to give continuous attention to the pandemic [23], which has not been included in previous studies. Therefore, we fill the research gap by exploring our research question as follows:

*RQ: During the pandemic, what characteristics of the information conveyed in articles on WeChat official accounts can affect users’ information behavior?*

### 1.1. Theoretical Basis and Hypothesis Development

#### 1.1.1. Health Information Dissemination

Dissemination is a learned response to stress; in uncertain circumstances, dissemination activities are frequent [24]. Uncertainty in public health emergency includes three dimensions: global health uncertainty, which is a “gap” in existing scientific knowledge about the virus; public health uncertainty, which is the difficulty in determining the epidemiological risk distribution; and clinical uncertainty, which refers to whether effective treatment can be provided [25]. Uncertainty can trigger negative feelings, such as stress and anxiety [26], and may cause people to engage in various communication behaviors, such as actively seeking information [27]. Thus, information dissemination is usually frequent in uncertain situations, such as NCP [28]. At present, many researchers are identifying advantages in information dissemination on social media. For example, Moorhead [29] and Moon et al. [30] found that online health information promotes health communication and helps users make scientific decisions on Facebook, WebMD, and other platforms. Hazzam [31] and Benetoli et al. [32] proposed that information on WhatsApp, YouTube, and Facebook can also help health professionals refresh their knowledge; it is also useful for their career development.

With social media becoming an essential healthcare tool [33], physicians are required to become active on social media and interact with their peers, patients, and the entire health community [34]. Dubbed as the “next-generation medicine” [35], social media can be used by physicians and other health workers to edit and disseminate information as words, pictures, or videos.

On WeChat official accounts, health information is mainly spread as articles, which users read and share. In this uncertain situation, many accounts actively report the state of pneumonia and recommend many preventive measures, thereby attracting a large wave of followers [3]. These problem-oriented pieces of information can provide actionable guidelines to fulfill users’ needs to cope with the pandemic [36], causing WeChat official accounts to receive a remarkable attention.

#### 1.1.2. Users’ Information Behavior

Users’ behavior on social networks is the inclination to use social network services and various activities [37]. Researchers studied users’ information behavior on some popular social media platforms. For example, Bian et al. [38] found that the tendency to discuss promotional Lynch syndrome-related health information on Twitter shows users’ prevention awareness. Iftikhar et al. [39] clarified that health information on WhatsApp, Facebook, and Twitter can urge users to verify it on Google. Meanwhile, Gan [40] summarized three factors, namely, hedonic, social, and utilitarian gratifications, which affect the tendency of WeChat users to like a post.

Users’ information behavior is manifested everywhere on the Internet [41]. Their behavior on WeChat official accounts includes acquiring information, liking a post [40], and following an account. Different behaviors may reflect different inclinations. For example, reading an article shows users’ interest in a certain health theme [42]. Liking a post reflects their preference and appreciation [12,43]. After reading an article, users can like it to show their appreciation for the important message [23], and following accounts may indicate that users want to know what is being posted and their willingness to pay continuous attention [44].

However, to the best of our knowledge, few studies have focused on analyzing the influence of information on users’ information behavior to explore specific characteristics that satisfy WeChat users. Thus, this study aimed to address this issue.

For this purpose, we developed multiple and simple linear regression models. We chose the number of different types of articles and the aggregated number of headlines on NCP posted on the selected accounts in a 7-day period as independent variables (a total of seven) to denote the health information source and reflect the dissemination state. We also chose the number of new followers and likes in this period as dependent variables to represent users’ information behavior. Then, we analyzed the relationship between information and behavior in quantity. We selected the number of related articles because it is a critical indicator in evaluating information dissemination [45]. Besides, for the impact of content on liking behaviors, we chose all of the headlines on NCP which won more than 10,000 likes to conduct our content analysis. Information can affect users’ information behavior on other media [13,14,15,16,17,18,19,20,21,22]. We want to explore whether information conveyed in each type of articles posted on WeChat can play the role, impacting users’ following and liking behavior. Thus, on WeChat official accounts, we drew the following hypotheses—*H1* to *H3*. These articles will be classified into different types in the later part of this paper.

*H1*: *The number of different types of articles on NCP could contribute to the change in the number of followers of an official account*.

*H2*: *The aggregated number of headlines on NCP could contribute to the total number of likes*.

*H3*: *The headlines with a great number of likes may possess common characteristics in content that can impact users’ liking behavior*.

## 2. Materials and Methods

### 2.1. Data Collection

**Figure 1:**
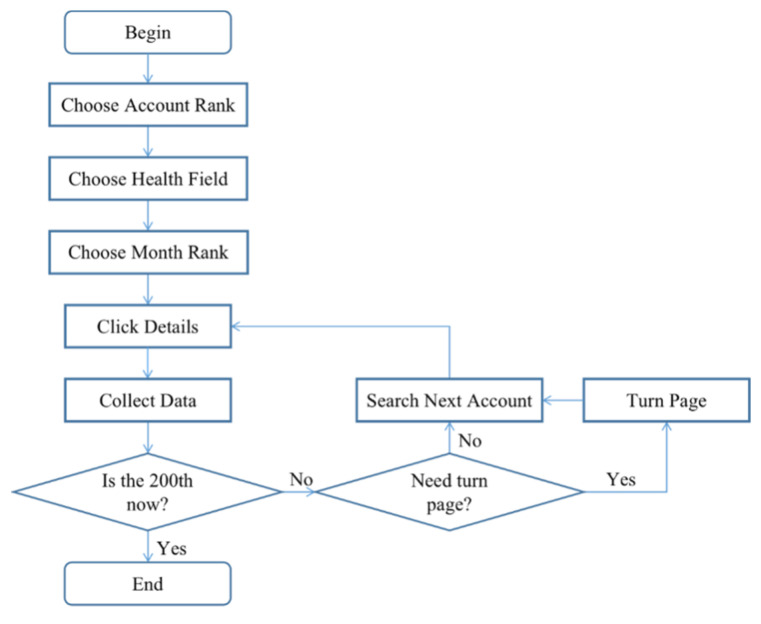
Data collection process.

**Figure 2:**
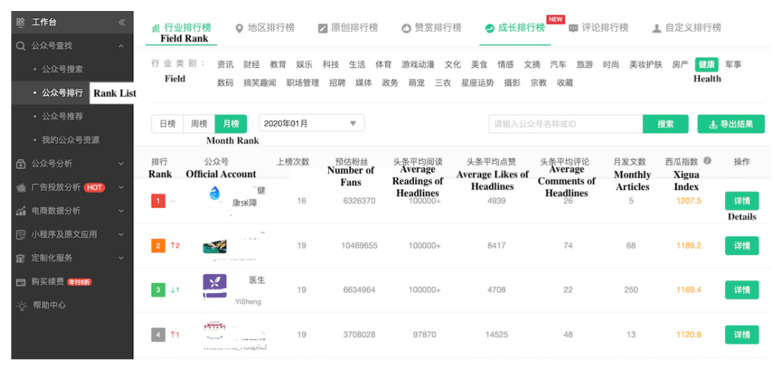
Screenshot of the rank list page. Some important nouns and items were translated into English. From: Xigua Data. Rank of Official Accounts. Available online: http://data.xiguaji.com/Home#/Rank/IndustryNew?Period=30&tid=4 (accessed on 10 April 2020).

**Figure 3:**
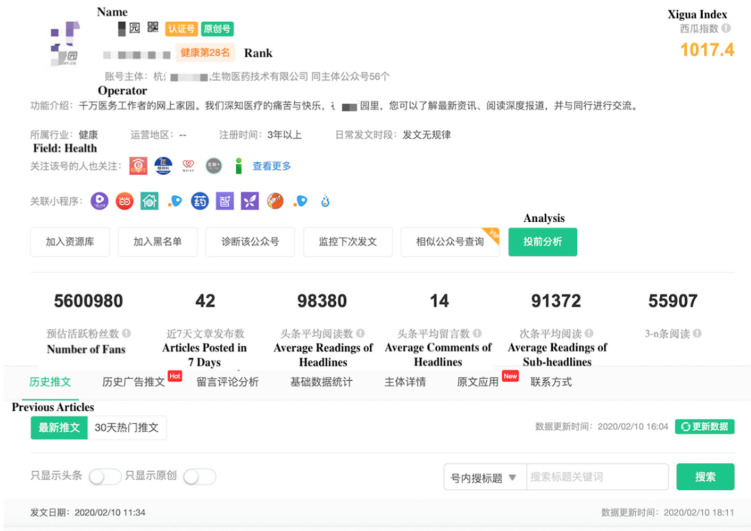
Screenshot of the data collection page. Some important nouns and items were translated into English. From: Xigua Data. Detailed Data of Official Accounts. Available online: http://data.xiguaji.com/Home#/Rank/IndustryNew?Period=30&tid=4 (accessed on 10 April 2020).

We collected data from the Xigua website (data.xiguaji.com) in China. It is a big data platform that provides operational data on WeChat official accounts. The data include the number of articles posted in the last 7 days, comments, the number of likes, and other information on each account. Xigua is an open-access website for researchers, and official accounts on this website can be classified into different fields, such as economy, sports, and health. We focused on health and used data on monthly rankings. We used Bazhuayu, a Chinese web crawler software for data collection, to collect data within the top 200 accounts as shown in Figure 1. The outbreak of the disease in China occurred on 19 January 2020. Since then, information regarding the pandemic has attracted considerable attention. At this time, the online reaction of the public may be greatly intense as they were faced with this severe condition suddenly. In a short period, their behaviors may be easier and more obvious to observe than before. Thus, we selected 21 January 2020, and 27 January 2020, as two time nodes to collect and classify account information, including the name, rank, operator, and number of followers. These accounts can be classified into three types based on their operators: nonmedical institution, medical institution, and individual accounts. Different types of accounts are operated by different stakeholders. Nonmedical institution accounts are operated by companies and governments; medical institution accounts are administrated by hospitals, including maternal and child care service centers; and individual accounts are managed by individuals. Table 2 presents the number of accounts. Then, we calculated the change in followers in 21–27 January 2020. Because we intended to study information on NCP, we filtered several accounts to identify the influence of information on the pandemic and deleted those who did not post any article related to NCP. For the 124 remaining accounts, 66.1% (82/124) were nonmedical institution accounts, 24.2% (30/124) were medical institution accounts, and 9.7% (12/124) were individual accounts. Figure 2 and Figure 3 show the screenshots of the rank list and data collection page, respectively.

### 2.2. Variables

Following an account and liking a post can represent users’ activity. The change in the number of followers in the 7-day period and the aggregated number of likes in the headlines that are correlated with NCP can reflect users’ information behavior. Thus, we used them as two dependent variables.

**Figure 4:**
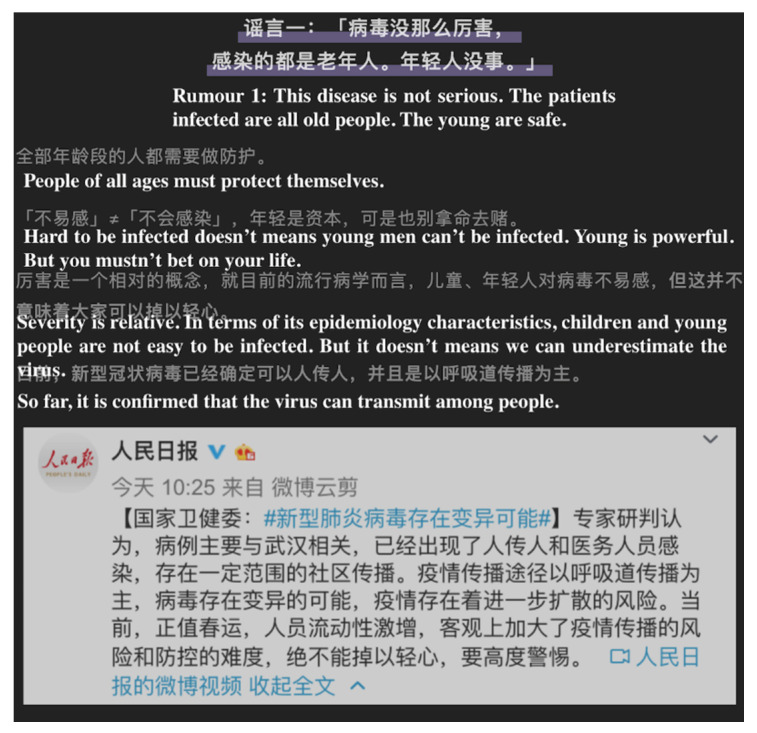
Screenshot of a counter-rumor article. From: DingXiangYiSheng. Novel coronavirus fears alcohol and high temperature, but vinegar, saline, and smoke are useless: 11 rumors you need to know. Available online: https://mp.weixin.qq.com/s?__biz=MjA1ODMxMDQwMQ==&mid=2657273064&idx=1&sn=c33004970adc91f70f2f42612aca6f63&chksm=4906c0867e714990e67d5473c373688c6346da7ff39ace49a111c9e72b2dca039d785dc4e475#rd (accessed on 31 April 2020).

**Figure 5:**
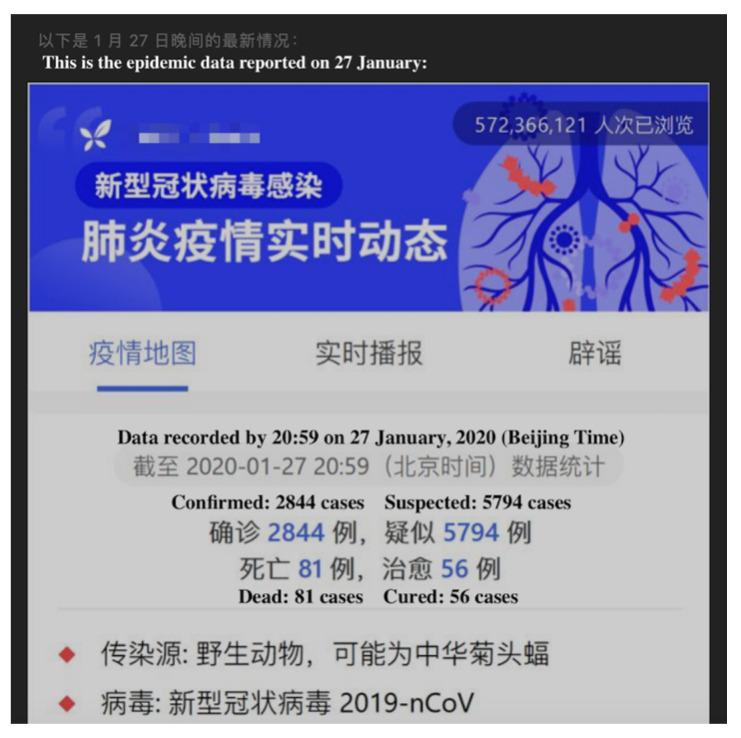
Screenshot of a report article. From: DingXiangYiSheng. COVID-19 Situation Report on 27 January 2020. Available online: https://mp.weixin.qq.com/s?__biz=MjA1ODMxMDQwMQ==&mid=2657273342&idx=2&sn=a5eb17c4e762d56bc471a5007526f090&chksm=4906c1907e714886d4a2d455fb377077727cedf68d7cffb47be2bd2641e45d4f3e99bb2d7112&scene=27#wechat_redirect (accessed on 31 April 2020).

**Figure 6:**
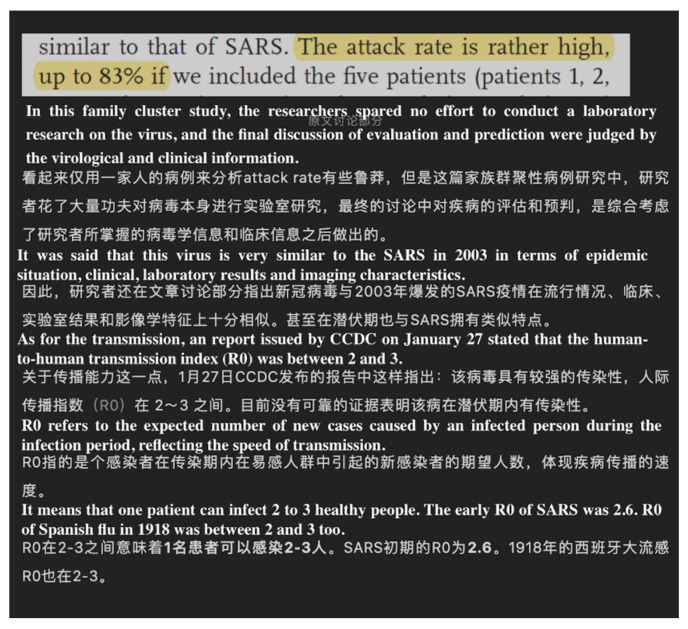
Screenshot of a science article. From: JieDi. What do you think of The mortality rate of the first 41 infected cases reached 15%? The Chinese CDC report is here. Available online: https://mp.weixin.qq.com/s?__biz=MjM5MDc3NjQwMA==&mid=2926680516&idx=4&sn=5510955bfd633041960d364d6a99abfe&chksm=8d3a94fbba4d1ded116d78153c87dcc4c6ed1554927187aab9af452fdf1b4937a5c66bec7144&scene=27#wechat_redirect (accessed on 31 April 2020).

**Figure 7:**
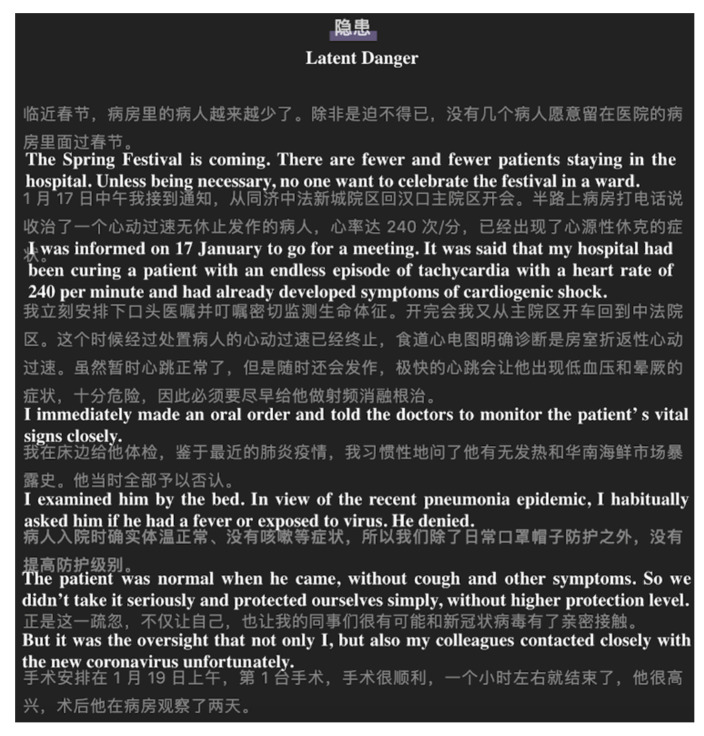
Screenshot of a story type. From: Ning Zhou. A Wuhan doctor was suspected of being infected. He recovered after 4 days’ isolation at home! Please spread his life-saving strategy to everyone. Available online: https://mp.weixin.qq.com/s?__biz=MzAxODI5OTI5Nw==&mid=2655524012&idx=1&sn=1db56d18ca7d7dd5870c24000cda8fde&chksm=8064d462b7135d74ddbaf7276f6eb9695bdde518acde13672f74bb7f4a34152f4a8f615e50a4&scene=27#wechat_redirect (accessed on 30 April 2020).

**Figure 8:**
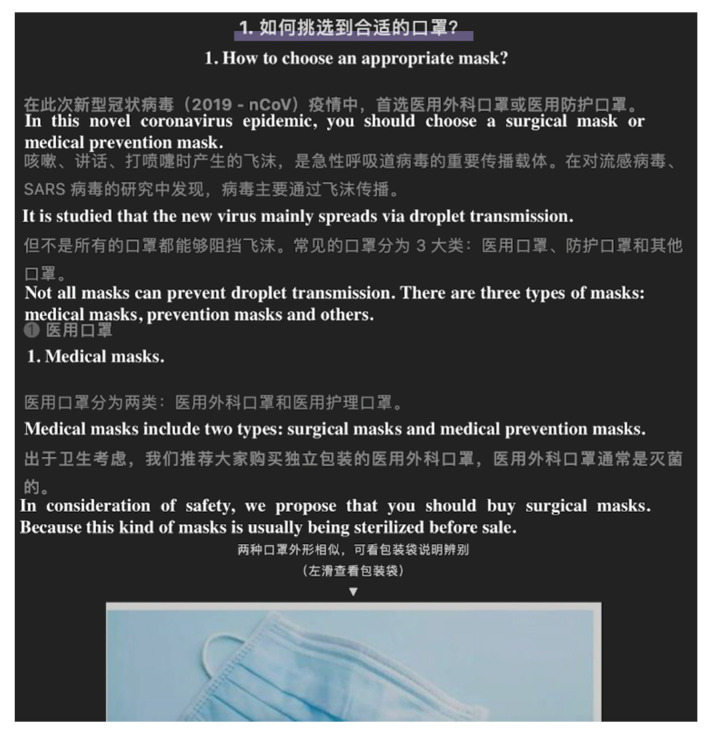
Screenshot of an instruction-type article. From: DingXiangYiSheng. Don’t put your mask casually! Here are the most detailed instructions on wearing your mask. Available online: https://mp.weixin.qq.com/s?__biz=MzA4MTg0MjA5OQ==&mid=2654891799&idx=1&sn=dba5017feccdfbc5d15e882ad6b75235&chksm=844415cbb3339cddab159f656e99a3d26ccea6339607ffa153d96f6173f762277c9f2495ef5f#rd (accessed on 31 April 2020).

**Figure 9:**
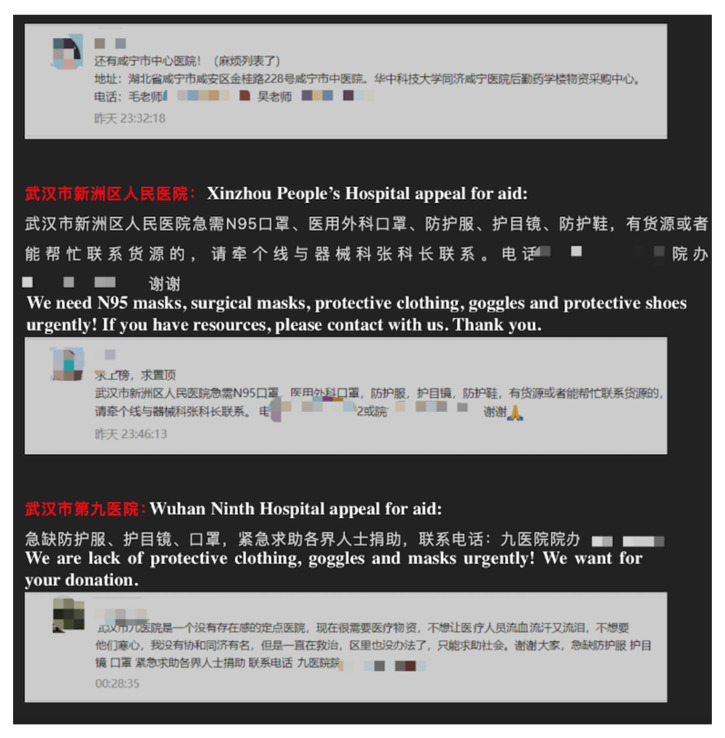
Screenshot of other types of articles. From: YiXueJie. Except for 18 tertiary hospitals in Wuhan, there are still many hospitals in Hubei appealing for help. Please share this article. Available online: https://mp.weixin.qq.com/s?__biz=MzU4MjQzNjAzMA==&mid=2247506900&idx=3&sn=554bd0369ebbbbe2179429ad83687e5a&chksm=fdbace05cacd47134ad603acd04c7cf3bcaf61399ace645442aa0095c8b37596e1f82635e32b&scene=27#wechat_redirect (accessed on 31 April 2020).

We recorded the state of articles on every account and counted the number of posts on NCP. We classified them into six types; namely, counter-rumor, report, science, story, instruction, and others. We classified articles that struck sensationalism or misinformation and clarified a fact as a counter-rumor article. We classified articles on news about the state of the pandemic, several facts, or a press conference conducted by the National Health Commission or other governmental institutions as a report. We also grouped an interview with professionals as a report. We categorized posts on scientific outcomes about NCP, explanations of this new virus, or information about psychology under science. We identified shared self—or public—description articles about how physicians resisted the pandemic in hospitals as a story. We identified posts that instructed the public to protect themselves or published a diagnosis and treatment guideline as instruction. Other posts, such as commentary, appealing for aid, advocating, and encouraging articles, were grouped under others. We classified posts that integrated more than one type of topic based on titles and main contents. This classification standard was approved by all the authors. We defined six independent variables for the six article types. Figure 4, Figure 5, Figure 6, Figure 7, Figure 8 and Figure 9 show six examples of different article types.

Moreover, we counted the number of headlines on NCP to explore its correlation with likes. Each account will post many articles on NCP every day, and headline is the first one with a conspicuous title and illustration. We recorded and counted the total number of likes in each article in this period. We defined headlines as another independent variable. Table 3 presents the collected and processed sample data. The scale of change in the number of followers was 10,000; for the number of likes, the scale was 1000.

### 2.3. Test of Normality

Before estimating the models, we tested whether the variables were normal. We used a one-sample Kolmogorov–Smirnov test to examine the normality of variables. Table 4 shows the results. The sample size was 124 (*N* = 124). All *p* values were below 0.001. Therefore, all variables were normal and could be estimated in the linear regression models.

### 2.4. Model Fitting

We developed a multiple linear regression model to explore the relationship between the change in the number of followers and the six types of articles. Meanwhile, we developed a simple linear regression model for the aggregated number of likes. We proved the normality of variables. Models are shown in the following two equations. *Y_i_* represents the change in the number of followers in the 7-day period. *Counter-rumor_i_*, *Report_i_*, *Science_i_*, *Story_i_*, *Instruction_i_*, and *Others_i_* denote the number of counter-rumor, report, science, story, instruction, and other types of articles, respectively. *Y_i_’* represents the aggregated number of likes in headlines in this period. *Headlines_i_* indicates the total number of headlines related to the pandemic.

(1) $$Y_{i}=\alpha_{0}+\alpha_{1}Counter-rumor_{i}+\alpha_{2}Report_{i}+\alpha_{3}Science_{i}+\alpha_{4}\;Story_{i}+\alpha_{5}\;Instruction_{i}+\alpha_{6}Others_{i}+\varepsilon_{i}$$

where *i* = 1,2,…, *n* index all accounts; *α*_0_ to *α*_6_ are the parameters to be estimated; *ε_i_* is the corresponding residue.

(2) $$Y_{i}^{’}=\beta_{0}+\beta_{1}Headlines_{i}+\varepsilon_{i}$$

where *i* = 1,2,…, *n* index all accounts; *β*_0_ and *β*_1_ are the parameters to be estimated; *ε_i_* is the corresponding residue.

### 2.5. Content Analysis

We designed our research to figure out the effect of information quantity on users’ information behaviors. We were also interested in the effect of content. We found an interesting phenomenon that among the accounts whose articles were usually unpopular, one article received a large number of likes. It did not correspond to the popularity of the account. For example, West China Hospital lagged in the rank list, and most of its posted headlines were plain. Nevertheless, on 22 January 2020, it posted a headline that received 60,225 likes, which might be a crucial factor that can affect our regression result. We can hardly find an article that received such an unexpected number of likes. We determined the reason why these articles were exceptional in terms of users’ liking behavior. To further conduct our study, we browsed the selected headlines that received more than 10,000 likes in this period and explored their characteristics in terms of content that could affect the liking behavior. We examined a total of 13 headlines. We code them from 4 perspectives: the account group that an article comes from, original/non-original articles, the article type, and the length of articles. Meanwhile, we recorded the form of multimedia applied in each article to show information (including the number of videos, pictures and graphics). The codebook is presented in Table A1, Table A2, Table A3 and Table A4 in Appendix A. The intercoder reliability was tested to be ideal. The coding results and some statistics are shown in Table 5.

## 3. Results

### 3.1. Results of Multiple Linear Regression

We used SPSS 25.0 to analyze the data. Table 6 shows the estimation results based on the least squares method and stepwise regression. We developed Model 1–3 to represent nonmedical institutions, medical institutions, and individual accounts, respectively. However, not all models could fit well. For nonmedical institution accounts in Model 1, the variables of report and story types had a significant effect (B = 2.724, *p* = 0.007; B = 14.875, *p* = 0.003) and played a positive role. The remaining variables were insignificant. For the medical institution accounts in Model 2, the variables of report and science types were significant (B = 4.381, *p* = 0.009; B = 31.564, *p* < 0.001) and positive. However, for individual accounts in Model 3, we did not obtain any result. Model 1 and 2 had adjusted R^2^ of 0.355 and 0.452, respectively, denoting an acceptable fit. We were unable to obtain a satisfactory result for Model 3. Thus, we partially confirmed H1.

### 3.2. Results of Simple Linear Regression

This section explored *H2*. Table 7 shows the simple linear regression result based on the least squares method and stepwise regression. Among the three groups, only nonmedical institution accounts in Model 1 showed significance. The variable of headlines played a positive role (B = 3.084, *p*< 0.001). The adjusted *R*^2^ was 0.317, denoting an acceptable fit. We did not discover significance for medical institution and individual accounts. Thus, *H2* was partially confirmed.

### 3.3. Results of Content Analysis

We found some impact factors of information dissemination on behavior, but we did not obtain a significant result in Model 2 and 3 when we analyzed headlines and likes. It may because of some exceptional articles with a large number of likes recorded in Table 5 that led to insignificance when we analyzed Model 2. When we discarded this datum, the analysis result was significant. Thus, this factor could remarkably affect our results. Some findings in content analysis are as follows.

#### 3.3.1. Accounts of Nonmedical Institutions Are More Preferred than Other Groups, and Dingxiang Doctor Is the Most Active

Of the 13 articles, 8 (62%) were posted by nonmedical institution accounts. Of these 8 articles, 5 were posted by Dingxiang Doctor, which was the most active account. Dingxiang Doctor was also the second-most popular in the rank list. Besides, one account named Dingxiang Yuan posted 1 article. These two accounts are affiliated with the same company called Hangzhou Lianke Meixun Biomedical Technology Corporation. In addition to these 8 articles, 1 (8%) was posted by medical institution accounts, and 4 (30%) were posted by personal accounts. However, the only article posted by West China Hospital received the most number of likes and reached 60,225. The reason why articles from the medical institution group accounted for the least proportion may be that these accounts usually post affairs about their affiliated hospitals, which may be less interesting in the public opinion. Compared with them, the public tend to prefer articles from nonmedical institution accounts. These accounts usually post various types of articles about common sense or short stories, which are easy for the public to understand and receive. This may be the reason why the public pay more attention to them and their articles.

#### 3.3.2. Original Articles May Not Contribute to Likes, but Instruction-Type Articles Are the Most Popular

Among the 13 articles, 7 (54%) were original, and 6 (46%) were not. We did not identify an evident preference for originality. In these headlines, instruction, story, others, and counter-rumor types accounted for 46% (6/13), 23% (3/13), 15% (2/13), and 8% (1/13), respectively. Report articles had the same proportion, accounting for 8% (1/13). For the two others, one article presented a timeline since the pandemic broke out, whereas the other article revealed several latent dangers after the city was locked down. Story-type articles were confirmed to be positive in regression analysis. An instruction-type article could provide suggestions during a public health emergency. This article type might be the most popular because it met users’ demands to seek prevention. Wu [46] believed that perceived usefulness is a precondition of users’ overall satisfaction. Perceived usefulness can affect users’ attitude and determine the continuance of using an information system [47]. Reading behavior can show the perceived usefulness from users [48], and liking an article may denote their gratifications [40]. Instruction articles will inspire users’ perceived usefulness and promote an account’s popularity.

#### 3.3.3. Length of Articles and Form of Information Can Enlighten Account Operators to Improve Their Performance

We studied the length of each article and the method of transmitting information in Table 5. The length varied among headlines; 8 articles in the 13 (62%) was coded as “2”, possessing 2000–4000 characters. Many articles limited the content length by using visual aids. For example, all articles applied infographics, including images and graphics. The post “*Can you go out without a mask? Experts recommended the proper wearing of masks.*” by West China Hospital had the most images (up to 28). Infographics and other visual aids, such as videos, can promote health information communication [49]. Using visuals based on conventional text gains an ideal outcome from the perspective of health information promotion [50]. Infographics and videos can help users visualize information and facilitate the straightforward understanding of information. As a result, the content is concise and clear, and it may help account operators to improve their performance in dissemination, making it easier for the public to receive information.

#### 3.3.4. Diversity in Types Can Enhance the Practicability of Articles, but Most Possess a Common Dimension

Although the types of articles varied, most of them integrated different types. For example, the article “*Can you go out without a mask? Experts recommended the proper wearing of masks.*” not only provided an instruction but also appended a report on the pandemic. The counter-rumor article “Novel coronavirus fears alcohol and high temperature, but vinegar, saline, and smoke are useless: *11 rumors you need to know.*” also taught several prevention methods. None of the articles had only one type of content. Our coders classified the articles on the titles and main content. Diversity in types could simultaneously enhance the practicability of the content and meet users’ different demands. However, the main part of the article should be specific to prevent it from being misinterpreted.

We counted and recorded high-frequency words in these articles. They are shown in Table 8. Words occurring more than five times were listed in it. All the articles introduced general features about COVID-19, mentioning some same words, such as Pandemic, Doctor, Infection, and so on. Along with it, we found that different types of these articles all referred to one common dimension. That is the instruction. For example, words including Mask, Wash hands, and Isolation indicated instructions on how to protect the public. They existed in most of these articles. Besides, Arbidol Hydrochloride Capsules is a drug used to relieve the state of infected cases. This noun also showed up in different articles, introducing an instruction on selecting drugs. Most of them introduced instructions on prevention. It may because of the usefulness perceived by readers that helped these articles win a large number of likes.

#### 3.3.5. Effects on Readers Are Positive Frequently but Should Consider the Limitation

**Figure 10:**
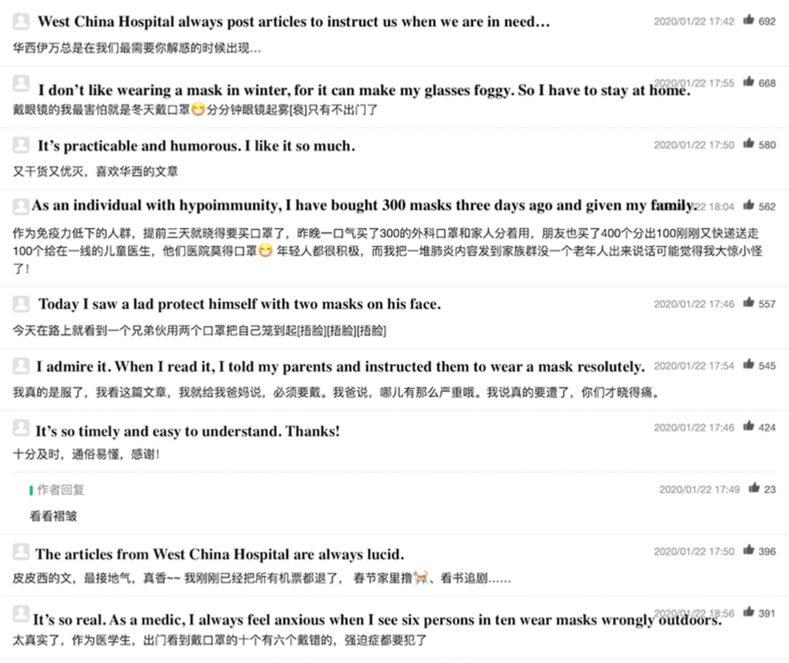
Screenshot of a review page. From: West China Hospital. Can you go out without a mask? Experts recommended the proper wearing of masks. Available online: https://mp.weixin.qq.com/s?__biz=MjM5Mjg4ODk0NQ==&mid=2652774509&idx=1&sn=798a83f859195aae2896260ef5e6cbe1&chksm=bd75e1738a026865444138f9d46dc8232eb65286f314fca37b25f103190064b1acc8298741a6#rd (accessed on 10 April 2020).

These articles usually have positive effects on readers. For example, Figure 10 shows the screenshot of reviews from readers of an article with the most likes. Most of the readers admired and appreciated the usefulness of the article. The comments suggested that the article could facilitate the timely acquisition of knowledge about prevention during a health crisis. Furthermore, a story about the heroic contributions of doctors and other people may inspire readers. A counter-rumor type of article may help users identify inaccurate information and prevent them from adopting inappropriate prevention methods. However, popularity is accompanied with limitations, and this issue should be considered. Given the severity reported in these articles, information may lead to unnecessary public panic. Some people even protect themselves excessively, as the disease is devastating if uncontrolled. However, with the contributions of physicians, we must be hopeful for the future situation. Therefore, the aspect of reducing the negative effects of articles on readers should be considered by account operators.

The organizational structure, location, and description of information on social media affect public attention [51]. Multimedia applications, such as infographics and videos, make the structure clear and concise. Certain types of articles can satisfy the public’s demand for information and improve the popularity of articles. Diversity in types can promote users’ liking behavior and health information dissemination because of the superior content of articles. Besides, these articles always referred to a common dimension which introduced some instructions. *H3* was proven to be true.

## 4. Discussion

### 4.1. Principal Findings

This study aimed to explore the effects of health information dissemination on users’ information behavior on WeChat official accounts. Our hypotheses were tested using two-wave data collected from the Xigua website over a period of 7 days. Meanwhile, we further explored the content characteristics based on the 13 headlines with an unexpected number of likes to answer our research question.

First, our results suggested that not all types of articles significantly affected the users’ tendency to follow an account. For nonmedical institution accounts, report and story types positively influenced the change in the number of followers. For medical institution accounts, report and science types exerted positive effects. However, we did not find significant relationships for individual accounts.

Second, the number of headlines on the pandemic contributed to likes for nonmedical institutions. However, we did not obtain the same result in medical institution and individual accounts. For medical institution accounts, we found an article with an unexpected number of likes of up to 60,225. When we rejected this information, the analysis result was significant. Thus, several articles with an unexpected number of likes could determine the regression result to a great extent.

Third, we reviewed 13 headlines to further explore the characteristics of information from articles that could affect users’ liking behavior. In the headlines, organizational structure, the manner of description, and the application of multimedia contributed to unexpected likes. Users showed an inclination to instruction and story types, especially those from nonmedical institution accounts. Health authorities should take advantage of these accounts to enhance health information dissemination and reduce public panic. Meanwhile, paying attention to methods of delivering messages was crucial, for the application of multimedia such as graphics, videos or pictures may make it easier for the public to understand and receive information. Besides, diversity in types was also crucial in encouraging likes, and the role of instructions should not be left out. These dimensions composed the common features of information that can impact users’ likes.

### 4.2. Theoretical and Practical Implications

This study has theoretical implications. On Facebook, Twitter, and other social media, information dissemination can affect users’ information behavior [31]. In the present study, we expanded the research scope to WeChat in China, especially in the health field. We identified several factors that affected users’ information behavior on this platform. For nonmedical institution accounts, report and story types of information should be emphasized. Likewise, report and science types should be promoted for medical institution accounts. Particular account groups, multiform transmission, and diversity in types, including instruction and story, are essential for promoting popularity.

This study also has practical implications. First, on social media, account operators can promote information dissemination. Analyzing users’ information behavior may allow them to determine the kind of information that satisfies the public. They should fully utilize the superiority of headlines to enhance diffusion. Second, the above conclusions could be explored further and in depth by analyzing Twitter, Facebook, or YouTube trends in other countries, contributing to the worldwide campaign in the medical informatics and health dissemination domains. This strategy might help authorities determine what kind of information the public needs. If dissemination is efficient, the public will receive accurate information and useful prevention suggestions in a timely manner. This method can help health authorities successfully manage this public health emergency [52].

### 4.3. Limitations

This research has several limitations. During the data analysis, we did not identify the significance of some variables because the sample size was small. For example, we only assessed 12 individual accounts among 124 accounts. For this reason, we could not ensure that such insignificant factors would not contribute to users’ information behavior. In our future work, we will involve more comments from users on different groups of accounts and expand the sample size to conduct further analyses.

We considered that likes mainly denote users’ appreciation. However, some readers may like an article without a valid reason or for conformity [53]. Other users can express their positive feelings by commenting. Thus, in further studies, we will assess the implications of users’ behavior, such as liking, following, commenting, and even sharing, based on quantitative methods. Interviews that reflect users’ actual feelings are also essential. We will also explore the effect of content on readers by analyzing their comments.

## 5. Conclusions

The effects of information dissemination on users’ information behavior during the COVID-19 pandemic were examined. Two models were developed to test our hypotheses that were partially confirmed. In content analysis, some common characteristics that contributed to users’ tendency to like a post were identified. However, this study has some limitations. In our future work, we will include more accounts and adopt measures such as developing a synthetic model and quantitatively assessing these behaviors to solve the problems.

## Figures and Tables

**Table 1:** Studies on the influence of health information on users’ behavior.

Information Behavior	Social Media	Conclusion	Reference
Seeking and Posting Behavior	YouTube Twitter Facebook Kakao Talk	Zika, MERS, and chikungunya messages motivate the public to search for related information frequently and to post actively.	Bragazzi et al. [13] Mahroum et al. [14] Jang et al. [15]
	Mommy Blog	Users with a personal connection to the health issue tend to post articles about it.	Burke-Garcia et al. [16]
Adoption and Sharing Behavior	MeetYou BabyTree Facebook	Pregnancy-related information influences expectant mothers to adopt and share from the perspective of perceived influence and prenatal attachment.	Zhu et al. [17] Harpel. [18] Lupton. [19]
Commenting Behavior	Microblog	Information correlated with the vaccine event or environmental health in China can significantly influence users’ comments.	An et al. [20] Wang et al. [21]
Prevention Behavior	Mommy Blog Instagram Facebook	Intervention messages on breast cancer can effectively affect prevention behavior and lead to high exposure scores in consideration of the influence of leaders’ opinion.	Wright et al. [22]

**Table 2:** Groups and numbers of official accounts.

Group	Number	Percentage
Nonmedical Institution	143	71.5%
Medical Institution	41	20.5%
Individual	16	8.0%

**Table 3:** Items and statistical variables.

Variable, Proxy, and Group	Minimum	Maximum	Mean (SD)
**Number of Followers** **Change in the Number of Followers in the 7-Day Period (10,000)**			
Nonmedical Institution	−18.26	271.41	18.31 (35.82)
Medical Institution	−15.92	90.85	9.96 (24.18)
Individual	−40.95	1021.84	110.73 (292.29)
**Number of Likes**			
**Aggregated number of likes in headlines on NCP in the 7-day period (1000)**			
Nonmedical Institution	0	159.25	5.30 (19.35)
Medical Institution	0	602.23	21.51 (109.70)
Individual	0.97	80.67	11.95 (23.27)
**Number of Articles Posted on NCP in the 7-Day Period**			
**Counter-Rumor**			
Nonmedical Institution	0	3	0.29 (0.62)
Medical Institution	0	1	0.07 (0.25)
Individual	0	2	0.50 (0.80)
**Report**			
Nonmedical Institution	0	19	2.20 (4.26)
Medical Institution	0	10	2.70 (3.31)
Individual	0	18	2.25 (5.14)
**Science**			
Nonmedical Institution	0	6	0.49 (1.10)
Medical Institution	0	2	0.43 (0.898)
Individual	0	2	0.25 (0.62)
**Story**			
Nonmedical Institution	0	4	0.30 (0.84)
Medical Institution	0	3	0.63 (1.33)
Individual	0	3	0.58 (1.00)
**Instruction**			
Nonmedical Institution	0	13	2.12 (2.96)
Medical Institution	0	3	2.13 (1.68)
Individual	0	9	2.25 (2.49)
**Others**			
Nonmedical Institution	0	7	0.72 (1.37)
Medical Institution	0	1	0.93 (0.83)
Individual	0	6	1.58 (1.93)
**Number of Headlines**			
**Aggregated Number of Headlines on NCP in the 7-Day Period**			
Nonmedical Institution	0	20	2.63 (3.58)
Medical Institution	0	5	1.23 (1.25)
Individual	1	6	3.75 (2.05)

**Table 4:** Results of one-sample Kolmogorov–Smirnov test.

Variables	*N*	Test Statistic	*p* Value
Change in the number of followers	124	0.329	<0.001
Aggregated number of likes	124	0.394	<0.001
Counter-rumor type	124	0.293	<0.001
Report type	124	0.376	<0.001
Science type	124	0.363	<0.001
Story type	124	0.487	<0.001
Instruction type	124	0.321	<0.001
Others	124	0.299	<0.001
Aggregated number of headlines	124	0.284	<0.001

**Table 5:** The coding results and statistics.

Title	Account Group	Originality	Type	Length ^a^	No. of Videos	No. of Pictures	No. of Graphics
A Wuhan doctor was suspected of being infected. He recovered after 4 days’ isolation at home! Please spread his life-saving strategy to everyone!	3	2	4	3	0	7	0
A doctor from Tongji Hospital described: Infected by virus and isolated for 4 days, what did I do?	1	2	4	3	0	7	0
Up to date! Wuhan Tongji and Wuhan Xiehe hospitals released a rapid diagnosis and treatment guideline for new coronavirus pneumonia!	1	2	5	3	0	1	0
Can you go out without a mask? Experts recommended the proper wearing of masks.	2	1	5	2	0	28	0
Battlefront doctors in Wuhan may fall down at any time.	1	2	4	2	0	6	0
How to stay at home safely during this pandemic? Doing 11 things well is enough.	1	1	5	2	0	3	0
Academician Zhong Nanshan said infection would exist among people! The novel coronavirus pneumonia is not as simple as you think! Repost to remind others!	3	2	5	2	1	23	1
Who delayed Wuhan?	1	2	6	2	0	1	0
Novel coronavirus fears alcohol and high temperature, but vinegar, saline, and smoke are useless: 11 rumors you need to know.	1	1	1	2	0	9	0
Origin of this pneumonia: a virus that has been put on the table by humans.	1	1	2	2	0	17	0
Since this pandemic, the most important control measure has not been taken seriously.	3	1	5	2	1	5	0
Pneumonia in Wuhan is not only as simple as sealing off the city! Share, and it is not too late to know!	3	1	6	1	0	5	0
A prevention guideline against the new pneumonia. Scientific prevention, we should not believe and transmit rumors.	1	1	5	1	0	16	0

^a^ Length: the number of characters in an article.

**Table 6:** Multiple linear regression result.

Variables	Change in the Number of Followers in the 7-Day Period (10,000) ^a^, Coefficient (95% CI)		
	Model 1 (*N* = 82)	Model 2 (*N* = 30)	Model 3 (*N* = 12)
**Independent Variables**			
Counter-rumor	−0.013	0.133	— ^d^
Report	2.724 ^c^ (0.782–4.666)	4.381 ^c^ (1.173–7.589)	—
Science	−0.210	31.564 ^c^ (16.751–46.377)	—
Story	14.875 ^c^ (5.057–24.692)	0.014	—
Instruction	0.121	0.212	—
Others	0.187	0.046	—
Constant	7.796 ^b^	−1.724	—

^a^ Model 1: adjusted *R*^2^ = 0.355; Model 2: adjusted *R*^2^ = 0.452. ^b^ *p* < 0.05. ^c^ *p* < 0.01. ^d^ Not included in the model.

**Table 7:** Simple linear regression result.

Variables	Number of Likes in the 7-Day Period (1000) ^a^, Coefficient (95% CI)		
	Model 1 (*N* = 82)	Model 2 (*N* = 30)	Model 3 (*N* = 12)
**Independent Variables**			
Headlines	3.084 ^b^ (2.096–4.071)	— ^c^	—
Constant	–2.823	—	—

^a^ Model 1: adjusted *R*^2^ = 0.317. ^b^ *p* < 0.01. ^c^ Not included in the model.

**Table 8:** High-Frequency Words in each article.

Title	High-Frequency Words
A Wuhan doctor was suspected of being infected. He recovered after 4 days’ isolation at home! Please spread his life-saving strategy to everyone!	Isolation, Temperature, Infection, Treatment, Virus, Doctor, Pandemic, At home, Novel, Arbidol Hydrochloride Capsules, Wuhan, Patient, Tertiary, Colleague
A doctor from Tongji Hospital described: Infected by virus and isolated for 4 days, what did I do?	Isolation, Pandemic, Temperature, Infection, Treatment, Doctor, Virus, Novel, Arbidol Hydrochloride Capsules, Pneumonia, Confrimed, At home
Up to date! Wuhan Tongji and Wuhan Xiehe hospitals released a rapid diagnosis and treatment guideline for new coronavirus pneumonia!	Pneumonia, Virus, Patient, Infection, Fever, Respiratory tract, Pandemic, Treatment, Viral, Flu, Corona, Arbidol Hydrochloride Capsules, Per os, Drag, Symptom, Relieve, Clinical, Amoxicillin
Can you go out without a mask? Experts recommended the proper wearing of masks.	Mask, Medical, Virus, Droplet transmission, Wear, Huaxi Hospital, Corona, Prevention, Infection, Wash hands, Contact, Filter, Sneeze, Cough
Battlefront doctors in Wuhan may fall down at any time.	Mask, Fever, Doctor, Clinic, Protection, Patient, Pandemic, Wuhan, Confirmed, Front-line, Emergency treatment, Infection, Respiratory tract, Expert
How to stay at home safely during this pandemic? Doing 11 things well is enough.	Mask, Wash hands, Protection, Virus, Cough, Sneeze, Contact, Infection, Wear masks, Dispose, Fever
Academician Zhong Nanshan said infection would exist among people! The novel coronavirus pneumonia is not as simple as you think! Repost to remind others!	Mask, Corona, Pneumonia, Virus, Novel, Infection, Protection, Tertiary, Wuhan, Infect among people, Confirmed Cases, Medical, Treatment, Contact, Prevention, Wash hands, Isolation
Who delayed Wuhan?	Wuhan, Virus, Mask
Novel coronavirus fears alcohol and high temperature, but vinegar, saline, and smoke are useless: 11 rumors you need to know.	Novel coronavirus, Rumor, Prevention, Infection, Mask, Pandemic, Pneumonia, Wuhan, Respiratory tract, Dingxiang Yuan, Wear masks, Saline, Treatment, Doctor, Zhongnan Shan
Origin of this pneumonia: a virus that has been put on the table by humans.	Virus, Wildlife, SARS, Corona, Seafood Market, Host, Manis pentadactyla, Paguma larvata, Dinner
Since this pandemic, the most important control measure has not been taken seriously.	Mask, Wash hands, Clean, Pandemic, Health, Virus, Policy, Protection
Pneumonia in Wuhan is not only as simple as sealing off the city! Share, and it is not too late to know!	Wuhan, Tertiary, Investigation, Lock down the city, Pneumonia, Corona, Patient, Fever, Virus
A prevention guideline against the new pneumonia. Scientific prevention, we should not believe and transmit rumors.	Coronavirus, Pneumonia, Protection, Prevention, Infection, Transmission

## Appendix A. Cookbook

**Table A1:** The account group that an article comes from.

Account Group	No	%
1 = Nonmedical Institution	8	62%
2 = Medical Institution	1	8%
3 = Individual	4	30%
9999 = NaN	0	0
Total	13	100%

**Table A2:** Original/ Non-original articles.

Originality	No	%
1 = Original	7	54%
2 = Non-original	6	46%
9999 = NaN	0	0
Total	13	100%

**Table A3:** The article type.

Type	No	%
1 = Counter-rumor	1	8%
2 = Report	1	8%
3 = Science	0	0
4 = Story	3	23%
5 = Instruction	6	46%
6 = Others	2	15%
9999 = NaN	0	0
Total	13	100%

**Table A4:** The length ^a^ of the article.

Type	No	%
1 = Below 2000 characters	2	15%
2 = 2000–4000 characters	8	62%
3 = Above 4000 characters	3	23%
9999 = NaN	0	0
Total	13	100%

^a^ Length: the number of characters in an article.
